# An unwanted reaction by the use of Haloperidol in hyperkinetic delirium

**DOI:** 10.1007/s40520-020-01649-2

**Published:** 2020-07-15

**Authors:** Francesca Remelli, Maura Bugada, Giulia Matteucci, Mattia Brunori, Giordano Gianotti, Amedeo Zurlo, Stefano Volpato

**Affiliations:** 1 Department of Medical Sciences, University of Ferrara, Via Aldo Moro 8, 44121 Ferrara, Italy; 2 Geriatrics Unit, Azienda Ospedaliero-Universitaria di Ferrara, Via Aldo Moro 8, 44121 Ferrara, Italy; 3 Orthogeriatrics Unit, Azienda Ospedaliero-Universitaria di Ferrara, Via Aldo Moro 8, 44121 Ferrara, Italy

**Keywords:** Elderly, Hyperkinetic delirium, Haloperidol, Myoclonus, Pancytopenia

## Introduction

Delirium is the most common acute neuropsychiatric disorder in the elderly, characterized by alteration of multiple cognitive function, in particular attention, due to a widespread metabolic brain damage, direct consequence of an ongoing clinical problem or pharmacologic therapy; the formal definition is given by Diagnostic and Statistical Manual of Mental Disorders 5th Edition (DSM-5) [1]. The prevalence of delirium in a healthcare setting is high and ranges from 35% among patients admitted to medical departments to 74% of post-operative delirium in surgical units [2]. Furthermore, in relation to the clinical presentation, delirium is classified into different subtypes, hyperkinetic, hypokinetic and mixed.

The first recommended approach to delirium prevention and treatment is non-pharmacologic aimed to identify underlying medical or pharmacological causes. The pharmacologic approach should be reserved only to cases with severe agitation and not responsive to the non-pharmacological approach. Haloperidol is the standard antipsychotic therapy used in these cases, also because of the long medical experience with this agent. Haloperidol can be administrated orally, intravenously or intramuscular, and low-dose (0.5 – 1 mg) are suggested to manage severe psychomotor agitation. The most frequent neurological adverse effects (AEs) of Haloperidol are extrapyramidal symptoms, while rarely neuroleptic malignant syndrome has been described. Among the cardiovascular AEs, prolonged corrected QT interval on electrocardiogram, with a major risk of malignant arrhythmia, has often been reported.

In this report, we describe the case of two unusual AEs developed in an older patient with a hyperkinetic delirium after the administration of intramuscular Haloperidol.

## Case presentation

A 94-year-old woman, living at home with her family, was admitted to the Emergency Room with deep asthenia. She had the following clinical history: arterial hypertension, dyslipidemia, chronic heart failure (NYHA class II), chronic gastritis, hypothyroidism, chronic renal failure (stage III) with hydronephrosis due to a high-grade urinary bladder cancer. Before admission, the patient was dependent in both Basic Activities of Daily Living (BADL 1/6 —able to feed herself) and Instrumental Activities of Daily Living (IADL 2/8—able to handle medications and use the telephone); moreover, she needed mild assistance to stand and walk. At medical examination and neuropsychological assessment, she was alert and partially oriented in time, space, and person (SPMSQ 6 right answers out of 10). The vital signs were normal: blood pressure 140/80 mmHg, body temperature 36.2 °C, heart rate 67 bpm regular, respiratory rate 12 breaths per minute, peripheral oxygen saturation 98% without any respiratory support. Clinical signs of systemic dehydration (shrivelled skin with decreased turgor, dry oral mucous membranes, and sunken-appearing eyes) were present. The remaining physical examination was normal. At admission, the plasma osmolality was 319 mOsm/kg with moderate hyponatremia and high levels of serum creatinine and urea; the urine specific gravity was increased (1.040 unit of measure). Additional laboratory data at admission and during a hospital stay are shown in Table.1. Chest and abdominal X-rays and abdominal-US did not reveal any abnormalities, except to the known bladder cancer. Brain CT without contrast showed generalized atrophy, mostly of frontal lobes; no acute lesions were described. The diagnosis was an acute exacerbation of chronic renal failure due to severe dehydration. The most likely cause of dehydration was the contemporary presence of chronic therapy with thiazide diuretics and very low oral water intake, common in older patients with cognitive impairment and physical disability. The estimated total water deficit was 2.8 L, so intravenous fluid therapy was started with crystalloid solutions (0.9% NaCl solution 1000 ml in 24 h/per day); chronic medications (antihypertensive drugs, PPIs, Levothyroxine) were confirmed, except to thiazide diuretics.

**Table.1:** Laboratory tests

	Admission	5° day	8° day	14° day
Haemoglobin (g/dL)	9.8	8.3	8.7	10.6
WBC (cells/mm^3^)	7.360	4.200	3.220	9.920
PLT (cells/mm^3^)	406.000	27.000	12.000	294.000
Creatinine (mg/dl)	3.81	2.84	–	1.23
Urea (mg/dl)	135	98	–	–
Sodium (mmol/mol)	129	147	–	142
Potassium (mmol/mol)	3.8	4.2	–	3.6
Glucose (mg/dl)	98	–	–	85
Osmolality (mOsm/kg)	319	–	–	–
Bilirubin	0.41	–	–	–
ALT	18	–	–	–
INR	1.14	1.05	–	–
CRP (mg/dl)	6.22	–	–	–
PCT (ng/ml)	–	1.23	0.18	–
Urine specific gravity	1.040	–	–	–

*CRP* C-reactive protein, *WBC* white blood cells, *PLT* platelets counts, *PCT* procalcitonin

During the first night of hospitalization, the patient developed severe agitation interpreted as hyperkinetic delirium, unresponsive to non-pharmacologic interventions, as reorientation, early mobility and family participation. Low-dose of intramuscular Haloperidol (1 mg) was administrated with partial clinical response. Few hours later, the patients developed rapid and involuntary movements of the four limbs with motor restlessness and inability to remain relaxed. 2 mg of intravenous lorazepam were administrated with transient recovery. In the suspect of seizures, electroencephalogram was performed that resulted negative for epileptic abnormalities. A diagnosis of drug-induced myoclonus was suspected and an oral treatment with Clonazepam (0.3 mg × 3/day) was started with a good clinical response.

Moreover, during hospitalization, a transient pancytopenia was found at blood tests, characterized by mild leukopenia (WBC = 3.200/μl), moderate anemia (haemoglobin = 8.7 g/dl) and severe thrombocytopenia (PLT = 12.000/μl). The patient did not develop any mucocutaneous petechiae or major bleeding; only one episode of modest and self-limiting nosebleed was observed. Serum levels of folic acid, vitamin B12, LDH, haptoglobin, procalcitonin and coagulation parameters were normal; the peripheral blood smear was not pathological. During hospital stay, heparin was never administered. Pancytopenia was treated with platelets and RBC transfusions and intravenous steroids, in the initial suspect of immune-mediate disease. In the following days, progressive normalization of laboratory tests occurred, including a reduction of serum creatinine and increase in platelet count and haemoglobin level up to physiologic values. Based on blood tests and the temporal association between the administration of Haloperidol and the hematological changes, we hypothesized drug-induced pancytopenia as the most likely diagnosis.

The patient was discharged from the Geriatric Unit after 14 days of hospitalization. The patient is still alive after 30 days.

## Discussion

As previously pointed out, the administration of Haloperidol can lead to many AEs, in particular extrapyramidal effects, including myoclonus (1–10% of cases), and more rarely to hematopoietic disorders, such as pancytopenia, leukopenia, thrombocytopenia (< 1% of cases). Myoclonus is characterized by brief, shock-like, involuntary movements caused by muscular contractions or inhibitions. The exact pathogenesis of antipsychotic-induced myoclonus is not completely clear, but the involvement of serotonergic, dopaminergic, and GABAergic mechanisms has been suggested. In particular, it is possible that the Haloperidol-induced effects on the serotonin system are due to a direct effect of the dopaminergic system on serotonergic pathways. Similarly, in the pathogenesis of pancytopenia the specific role of Haloperidol is still not clear, although some information is available about the mechanisms of drug-induced immune thrombocytopenia: it has been hypothesized to be either decreased platelet production or increased peripheral destruction.

Comparing our case with other reports described in the literature with similar clinical presentations, we have selected 2 cases of myoclonus [3, 4] and 1 case of thrombocytopenia provoked by Haloperidol administration [5]. To the best of our knowledge, our case is the first describing 2 important side effects simultaneously occurring after administration of the first dose of Haloperidol. In fact, concerning both myoclonus and thrombocytopenia, in the cases previously described in the literature, the disorder occurred after repeated administrations of Haloperidol [3, 4] or after treatment with three different neuroleptics and multiple doses [5] respectively. Moreover, also the time of development of myoclonus was different: our patient developed the disorder just a few hours after Haloperidol administration, while in the mentioned cases it occurred some days later [3, 4].

It is also important to highlight that in our clinical case, the patient has been never treated with any of other classes of drugs, except to antipsychotics, that potentially can cause myoclonus (in particular opiates, antidepressants and antibiotics). We have also ruled out that myoclonus was not a consequence of a brain impairment due to hyponatremia, because this was moderate, and the neurological disorder occurred just after the administration of Haloperidol.

About the development of thrombocytopenia, the most reasonable assumption is that it was caused by Haloperidol administration, because of the absence of other possible causes (other myelotoxic medications, sepsis, concurrent hematopoietic or other systemic disorders) and rapid normalization of platelet value after transfusion and drug withdrawal. Moreover, there are no other available case reports that described Haloperidol induced pancytopenia.

The reasons why our patient developed both of these AEs could be multiple. In addition to presumable individual predisposition and older age, another risk factor was the contemporary presence of dehydration and exacerbation of chronic renal failure that increases the blood concentration of the drug. Furthermore, the patient was affected by hypothyroidism and the treatment with Levothyroxine has been demonstrated to facilitate the toxicity of Haloperidol.

The AEs diagnosis is often a diagnosis of exclusion. In our case, the temporal correlation between drug administration and occurrence of myoclonus and pancytopenia, the progressive clinical and laboratory improvement following drug withdrawal and absence of other possible causes strongly support the diagnosis of drug-induced neurological-hematological damages.

## Conclusion

We presented a case of myoclonus and pancytopenia induced by Haloperidol during an acute confusional state. Although in literature there is unanimous consensus to define non-pharmacological treatment as the best approach to manage hyperkinetic delirium, in clinical practice and acute care setting, the use of antipsychotics is still common, despite being affected by a number of severe adverse events especially in older patients. Although many adverse reactions are dose and time-dependent, many other can be unpredictable and a high suspicion is needed to make an early diagnosis, and implement immediate treatment, including withdrawal of the suspected medication. In clinical practice, iatrogenic genesis underlying any clinical or laboratoristic variation should always be suspected, especially in older people, who are extremely susceptible to drug-induced adverse events.
